# Surrounding vascular geometry associated with basilar tip aneurysm formation

**DOI:** 10.1038/s41598-020-74266-8

**Published:** 2020-10-21

**Authors:** Jian Zhang, Anil Can, Pui Man Rosalind Lai, Srinivasan Mukundan, Victor M. Castro, Dmitriy Dligach, Sean Finan, Vivian S. Gainer, Nancy A. Shadick, Guergana Savova, Shawn N. Murphy, Tianxi Cai, Scott T. Weiss, Rose Du

**Affiliations:** 1 Department of Neurosurgery, Brigham and Women’s Hospital, 75 Francis Street, Boston, MA 02115 USA; 2 Department of Neurosurgery & Brain and Nerve Research Laboratory, The First Affiliated Hospital of Soochow University, Suzhou, Jiangsu Province China; 3 Department of Neurosurgery, Amsterdam University Medical Centers, Amsterdam, the Netherlands; 4 Department of Radiology, Brigham and Women’s Hospital, Boston, MA USA; 5 Research Information Systems and Computing, Massachusetts General Brigham, Boston, MA USA; 6 Boston Children’s Hospital Informatics Program, Boston, MA USA; 7 Department of Computer Science, Loyola University, Chicago, IL USA; 8 Division of Rheumatology, Immunology and Allergy, Brigham and Women’s Hospital, Boston, MA USA; 9 Department of Neurology, Massachusetts General Hospital, Boston, MA USA; 10 Biostatistics, Harvard School of Public Health, Boston, MA USA; 11 Channing Division of Network Medicine, Brigham and Women’s Hospital, Boston, MA USA

**Keywords:** Neurology, Neurological disorders, Cerebrovascular disorders

## Abstract

Hemodynamic stress is thought to play an important role in the formation of intracranial aneurysms, which is conditioned by the geometry of the surrounding vasculature. Our goal was to identify image-based morphological parameters that were associated with basilar artery tip aneurysms (BTA) in a location-specific manner. Three-dimensional morphological parameters obtained from CT-angiography (CTA) or digital subtraction angiography (DSA) from 207 patients with BTAs and a control group of 106 patients with aneurysms elsewhere to control for non-morphological factors, who were diagnosed at the Brigham and Women’s Hospital and Massachusetts General Hospital between 1990 and 2016, were evaluated. We examined the presence of hypoplastic, aplastic or fetal PCoAs, vertebral dominance, and diameters and angles of surrounding parent and daughter vessels. Univariable and multivariable statistical analyses were performed to determine statistical significance. Sensitivity analyses with small (≤ 3 mm) aneurysms only and with angles excluded, were also performed. In multivariable analysis, daughter–daughter angle was directly, and parent artery diameter and diameter size ratio were inversely associated with BTAs. These results remained significant in the subgroup analysis of small aneurysms (width ≤ 3 mm) and when angles were excluded. These easily measurable and robust parameters that are unlikely to be affected by aneurysm formation could aid in risk stratification for the formation of BTAs in high-risk patients.

## Introduction

Although the pathogenesis of intracranial aneurysms (IA) has been studied extensively, it is still poorly understood. Aneurysm formation is likely multifactorial, involving genetic predisposition and environmental factors such has hypertension and tobacco use^1–3^. In addition to these factors, there is growing evidence that morphological factors of the surrounding vasculature may play a key role in intracranial aneurysm formation, by triggering focal degenerative mechanisms at the vessel wall due to hemodynamic stress^4–13^. In particular arterial bifurcations are believed to be susceptible to aneurysm formation^5^. However, studies investigating the contribution of vascular geometry on aneurysm formation at the basilar apex are limited in the small number of patients with aneurysms included (varying from 8 to 59 patients with basilar tip aneurysms (BTA)), the lack of control groups harboring intracranial aneurysms elsewhere in order to control for genetic predisposition, and the very limited number of geometric variables included ^5,14–16^. Here we present the largest study to date with 207 basilar tip aneurysms and 106 control patients with other intracranial aneurysms, in which we evaluated a wide variety of image-based morphological variables that correlate with BTA formation in a location-specific manner.

## Methods

### Patient selection

We identified patients diagnosed with an intracranial aneurysm using natural language processing (NLP) in conjunction with manual medical record review from the Partners Healthcare Patients Data Registry (RPDR), which includes 4.2 million patients who have received care from the Brigham and Women’s Hospital (BWH) and Massachusetts General Hospital (MGH) between 1990 and 2016. Using a machine learning algorithm on both codified and NLP data, 5,589 patients with potential aneurysms were identified^17^ of which 727 patients were also seen on clinical presentation from 2007–2013 with prospectively collected data. 474 additional patients with prospectively collected data who were seen on clinical presentation from 2013–2016, were also included. This resulted in a total of 6,063 patients, which were then manually reviewed by AC and RD, leading to a final number of 4,701 patients with definite saccular aneurysms. 207 patients with basilar artery aneurysms had available imaging of sufficient quality which were obtained using the mi2b2 open-source software to comply with research privacy requirements. An additional 106 patients with aneurysms in other locations who were selected randomly were included as a control (non-BTA) group in order to control for any non-morphological risk factors for aneurysm formation. We excluded patients with non-saccular (fusiform) aneurysms or aneurysms associated with arteriovenous malformations. Demographic and clinical information, including gender, age, tobacco and alcohol use, history of hypertension, and family history of intracranial aneurysms and subarachnoid hemorrhage, was retrieved from medical records. This study was approved by the Partners Institutional Review Board which waived the requirement for informed consent. All procedures performed were in accordance with the ethical standards of the institutional review board and with the 1964 Helsinki declaration and its later amendments or comparable ethical standards.

### Reconstruction of 3D models

Using preoperative CTA via the Vitrea Advanced Visualization software (version 6.9.68.1, Vital Images, Minnetonka, MN), three-dimensional (3D) models of aneurysms and their surrounding vasculature were generated. The software creates a spatial reconstruction of the vasculature from axial CTA images in the DICOM (Digital Images and Communication in Medicine) format. DSA studies with 3D reconstructions were evaluated directly. We manually measured lengths and angles. In order to ensure accurate measurements, windowing for the 3D reconstructions were validated against the multiplanar reconstructions.

### Definition of morphological parameters

**Figure 1:**
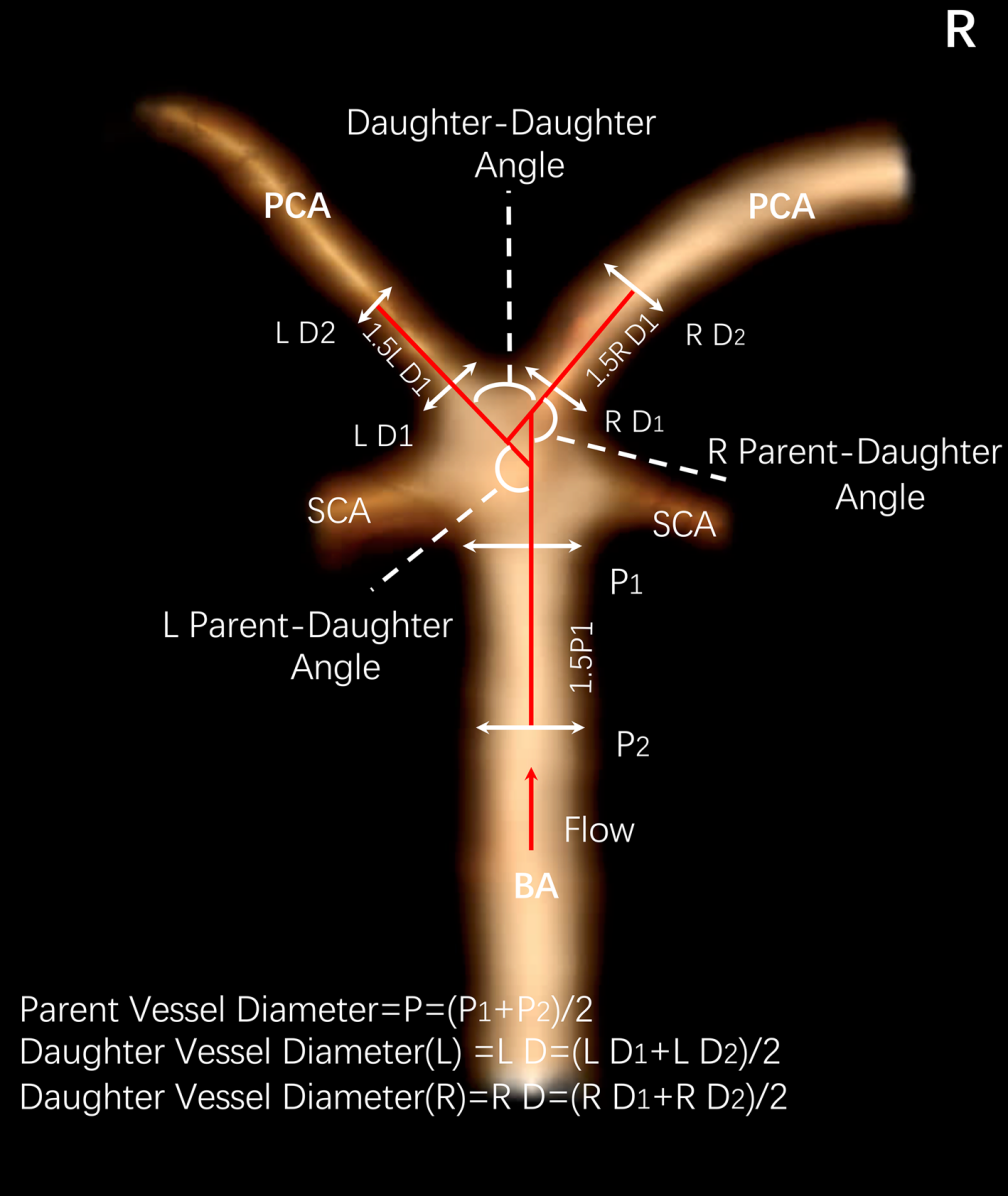
Illustration of morphological parameters.

We examined diameters and vessel-to-vessel angles of the main surrounding vessels around the basilar apex, including the distal basilar artery and P1 segment of the posterior cerebral arteries (PCAs) (Fig. 1). In case of hypoplastic or aplastic posterior communicating arteries (PCoAs) and/or fetal PCoAs, the number of vessels with this anatomical variation was noted (e.g. unilateral or bilateral). A PCoA was considered hypoplastic if its diameter was less than half of the contralateral PCoA. A PCoA was considered aplastic if it was not visible on CTA. Vertebral artery dominance was defined as the presence of unequal vertebral artery diameters. Vessel diameters were measured by averaging the diameter of the cross-section of a vessel (D) just proximal to the neck of the aneurysm and the diameter of the cross-section at 1.5 times D from the neck of the aneurysm. We calculated the average diameters of the parent (basilar) artery, larger daughter (P1) and the smaller daughter (P1) branches in this manner. The diameter size ratio was defined as the parent artery diameter divided by the sum of the diameters of both daughter branches, and the daughter diameter ratio was defined as the larger daughter artery diameter divided by the smaller daughter artery diameter. Daughter–daughter angle was defined as the angle formed between the daughter vessels and the parent–daughter angle ratio was defined as the larger angle between the parent vessel and the daughter vessel divided by the smaller angle between the parent vessel and the daughter vessel.

### Statistical analysis

Differences in baseline characteristics between the BTA and non-BTA groups were calculated using the *t-*test for continuous variables and the Pearson's chi-square test for categorical variables. Univariable and multivariable logistic regression models were used to test for effects of different morphological parameters on BTA presence, with a backward elimination procedure to identify significant confounders. Firth's bias-reduced penalized-likelihood logistic regression was used to account for the problem of complete separation. We used cut-off values of 0.1 in order to select the initial set of variables to be included in the initial multivariable model for backward elimination. Adjusted odds ratios (OR) with 95% confidence intervals (CIs) were calculated and p < 0.05 was considered significant. In order to control for the possibility that aneurysm formation could affect the surrounding vessel geometry (by increasing the P1–P1 angle for example), we included a subgroup analysis with small aneurysms only (≤ 3 mm) and a sensitivity analysis with the vascular angles excluded. Pearson’s correlation coefficient test was applied to assess the correlation between aneurysm width and daughter–daughter angle. All statistical analyses were performed using the Stata statistical software package (version 14, StataCorp. College Station, TX) and the *logistf*^18^ package in R^19^ (version 4.0.0).

## Results

Two hundred and seven patients with basilar tip aneurysms (BTA) and 106 patients with intracranial aneurysms elsewhere (non-BTA) were included in this study. Table 1 shows the demographic data and clinical risk factors of patients with BTA and non-BTA aneurysms. The mean patient age was 55.8 (12.0 SD), and 77.3% of patients were female. Patients with BTA aneurysms were significantly older than non-BTA patients. None of the other variables were significantly different between the two groups (Table 1).

**Table 1:** Demographic data and clinical risk factors of patients with and without basilar tip aneurysms (BTAs).

Variables	All patients N = 313	BTA N = 207	Non-BTA N = 106	P-value
Age (SD)	55.8 (12.0)	57.0 (11.3)	53.6 (13.2)	0.02
Female (%)	242 (77.3)	157 (75.8)	85 (80.2)	0.39
Alcohol use (current) (%)	150 (47.9)	96 (46.4)	54 (50.9)	0.49
Tobacco use (current) (%)	122 (0.39)	81 (39.1)	41 (38.7)	0.96
Hypertension (%)	160 (51.3)	110 (53.4)	50 (47.2)	0.30
Family history of SAH (%)	34 (10.9)	23 (11.1)	11 (10.6)	0.89
Family history of aneurysms (%)	65 (20.9)	40 (19.3)	25 (24.0)	0.34
History of ruptured aneurysm (%)	127 (40.6)	83 (40.1)	44 (41.5)	0.81

SAH = subarachnoid hemorrhage.

We then examined the predefined vascular geometry characteristics of the BTA and non-BTA groups (Table 2). Unilateral aplastic PCoAs were frequent in the BTA group (3.9% vs 0.0%). Mean basilar artery diameters were smaller in the BTA group (2.49 mm vs 2.64 mm) with smaller diameter size ratios (0.71 vs 0.80). Daughter–daughter angles were significantly larger in the BTA group compared to the non-BTA group (139.5 vs 95.5 degrees) and also parent–daughter angle ratios were larger (1.34 vs 1.15 degrees). Hypoplastic PCoAs, fetal PCoAs, vertebral dominance and daughter diameter ratios did not significantly differ between the two groups.

**Table 2:** Characteristics of the surrounding vasculature for basilar tip aneurysms (BTAs) and non-basilar tip aneurysms (non-BTAs) (N = 313).

Variables	All N = 313	BTA N = 207	Non-BTA N = 106	P-value
Hypoplastic PCoA (%)				
No	194 (62.0)	121 (58.5)	73 (68.9)	0.07
Unilateral	63 (20.1)	46 (22.2)	17 (16.0)	0.20
Bilateral	56 (17.9)	40 (19.3)	16 (15.1)	0.36
Aplastic PCoA (%)				
No	301 (96.2)	195 (94.2)	106 (100.0)	0.02
Unilateral	8 (2.6)	8 (3.9)	0 (0.0)	0.04
Bilateral	4 (1.3)	4 (1.9)	0 (0.0)	0.15
Fetal PCoA (%)				
No	287 (91.7)	190 (91.8)	97 (91.5)	0.93
Unilateral	20 (6.4)	12 (5.8)	8 (7.5)	0.56
Bilateral	6 (1.9)	5 (2.4)	1 (0.9)	0.36
Vertebral dominance (%)	70 (22.4)	43 (20.8)	27 (25.5)	0.35
Daughter diameter ratio (larger/smaller) (SD)	1.19 (0.26)	1.18 (0.22)	1.22 (0.33)	0.18
Basilar artery diameter in mm (SD)	2.54 (0.46)	2.49 (0.46)	2.64 (0.44)	0.01
Diameter size ratio (Parent/(D1 + D2))	0.74 (0.11)	0.71 (0.11)	0.80 (0.10)	< 0.01
Daughter–daughter angle in degrees (SD)	124.58 (29.03)	139.5 (21.6)	95.5 (17.3)	< 0.01
Parent–daughter angle ratio (SD)	1.27 (0.63)	1.34 (0.77)	1.15 (0.13)	0.01

D1 = diameter of daughter vessel 1, D2 = diameter of daughter vessel 2, PCoA = posterior communicating artery.

**Figure 2:**
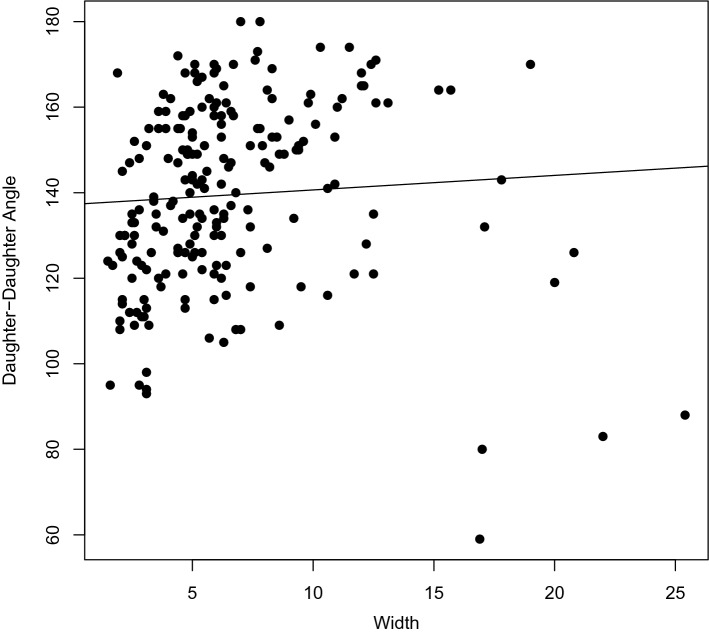
Plot of aneurysm width vs. daughter–daughter angle. Pearson’s correlation test for the association between aneurysm width and daughter–daughter angle was not significant (correlation = 0.06, p = 0.36).

Table 3 shows the results of the univariable and multivariable analyses for BTA-presence. In the univariable analyses, age (OR 1.02, 95% CI 1.00–1.05), daughter–daughter angle (OR 1.11, 95% CI 1.08–1.13), parent–daughter angle ratio (OR 24.94, 95% CI 5.86–106.1), and unilateral aplastic PCoA (OR 9.26, 95% CI 1.14–1201) were significantly associated with BTA presence. In contrast, parent artery diameter (OR 0.49, 95% CI 0.29–0.83) and diameter size ratio (OR 4.4 × 10^−4^, 95% CI 3.1 × 10^−5^–5.0 × 10^−3^) were significantly inversely associated with BTA presence. Gender, alcohol use, tobacco use, hypertension, family history of SAH or aneurysms, the presence of ruptured aneurysms, hypoplastic PCoAs, vertebral dominance and daughter diameter ratio were not statistically significant predictors of BTA presence. In multivariable analysis, daughter–daughter angle (OR 1.11, 95% CI 1.08–1.14) was significantly associated with BTA presence. In contrast, parent artery diameter (OR 0.18, 95% CI 0.06–0.46) and diameter size ratio (OR 0.001, 95% CI 2.76 × 10^−5^–0.05) were significantly inversely associated with BTA presence. When we removed the angle-related variables from multivariable analysis, parent artery diameter and diameter size ratio retained significance in the same direction. In a subgroup analysis of small aneurysms (width ≤ 3 mm), daughter–daughter angle was still significantly associated with BTA presence (OR 1.10, 95% 1.06–1.15), as were parent artery diameter and diameter size ratio. Pearson’s correlation test for the association between daughter–daughter angle and aneurysm width was not significant (correlation = 0.06, p = 0.36) (Fig. 2).

**Table 3:** Univariable and multivariable logistic regression for the presence of a basilar tip aneurysm (N = 313).

Variables	Univariable		Multivariable N = 313		Multivariable^a^ N = 139		Multivariable^b^ N = 313	
	OR (95% CI)	P-val	OR (95% CI)	P-val	OR (95% CI)	P-val	OR (95% CI)	P-val
Age	1.02 (1.00–1.05)	0.02	–	–	–	–	–	–
Female	0.78 (0.44–1.38)	0.39	–	–	–	–	–	–
Alcohol use (current)	0.85 (0.53–1.36)	0.49	–	–	–	–	–	–
Tobacco use (current)	1.01 (0.62–1.64)	0.96	–	–	–	–	–	–
Hypertension	1.28 (0.80–2.05)	0.30	–	–	–	–	–	–
Family history of SAH	0.76 (0.43–1.33)	0.34	–	–	–	–	–	–
Family history of aneurysms	1.06 (0.49–2.26)	0.89	–	–	–	–	–	–
Ruptured aneurysm	0.94 (0.59–1.52)	0.81	–	–	–	–	–	–
Hypoplastic PCoA								
Unilateral vs. no	1.63 (0.87–3.06)	0.13	–	–	–	–	–	–
Bilateral vs. no	1.51 (0.79–2.88)	0.21	–	–	–	–	–	–
Aplastic PCoA								
Unilateral vs. no	9.26 (1.14–1201)	0.03	–	–				
Bilateral vs. no	4.90 (0.52–652)	0.19	–	–				
Fetal PCoA								
Unilateral vs. no	0.77 (0.30–1.94)	0.57	–	–	–	–	–	–
Bilateral vs. no	2.55 (0.29–22.2)	0.40	–	–	–	–	–	–
Vertebral dominance	0.77 (0.44–1.33)	0.35	–	–	–	–	–	–
Daughter diameter ratio (larger/smaller)	0.56 (0.23–1.35)	0.20	–	–	–	–	–	–
Parent artery (basilar artery) diameter in mm	0.49 (0.29–0.83)	< 0.01	0.18 (0.06–0.46)	< 0.01	0.17 (0.03–0.73)	0.02	0.52 (0.29–0.93)	0.03
Diameter size ratio (Parent/(D1 + D2))	4.36 × 10^−4^ (3.06 × 10^−5^–0.005)	< 0.01	0.001 (2.76 × 10^−5^–0.05)	< 0.01	6.80 × 10^−6^ (2.65 × 10^−10^–0.04)	0.01	4.27 × 10^−4^ (2.87 × 10^−5^–0.005)	< 0.01
Daughter–daughter angle in degrees	1.11 (1.08–1.13)	< 0.01	1.11 (1.08–1.14)	< 0.01	1.10 (1.06–1.15)	< 0.01	–	–
Parent–daughter angle ratio	24.94 (5.86–106.1)	< 0.01	–	–	–	–	–	–

^**a**^Subgroup analysis with small basilar tip aneurysms only (width ≤ 3 mm).

^**b**^Sensitivity analysis without angles.

D1 = diameter of daughter vessel 1, D2 = diameter of daughter vessel 2, PCoA = posterior communicating artery, SAH = subarachnoid hemorrhage.

## Discussion

In this study, we showed daughter–daughter (P1–P1) angle to be associated with the presence of basilar artery tip aneurysms (BTAs), whereas parent artery diameter and diameter size ratio were inversely associated. Notably, the significance and direction of our results remained the same in the subgroup of small aneurysms (width ≤ 3 mm) and we found no correlation between daughter–daughter angle and the width of the aneurysm.

Intracranial aneurysm formation is associated with arterial wall deficiencies, and there is a growing evidence that wall shear stress (WSS)—a tangential frictional force exerted by flowing blood on the arterial endothelium—is a crucial element in intracranial aneurysm initiation ^20^. In line with our finding that parent artery diameter was inversely associated with the presence of BTAs, Farnoush et al. showed in a computation fluid dynamic simulation of cerebral bifurcation aneurysms that a smaller parent artery diameter leads to higher wall shear stress (WSS) and more energy loss at the apex of the bifurcation ^21^. Furthermore, in a recent meta-analysis, we showed a high positive correlation between high WSS and location of aneurysm formation ^22^. Although the exact mechanisms are unknown, it is believed that in response to high WSS, mechanical receptors in endothelial cells sense this increase in tension and respond by arterial dilatation, eventually leading to sustained degradation of extracellular matrix and consequent aneurysm formation ^23,24^. In addition, it is thought that due to the high WSS, endothelial cell signaling results in macrophage infiltration which disrupts the internal elastic lamina and the collagen matrix ^25^. It should be noted, however, that the absolute difference in the basilar artery diameter between the BTA and non-BTA group in our study is very small (0.15 mm).

Diameter size ratio, defined as the parent artery diameter divided by the sum of the diameters of both daughter branches, is unlikely to be changed by the formation of the aneurysm itself, thus providing a more robust measure of the relative relationship between the diameter of the basilar artery and the daughter vessels. Flow within the basilar bifurcation depends on various geometric considerations, including the relative caliber of the daughter and parent branches and the bifurcation angle, and is believed to follow the vascular optimality principle (VOP) ^26^. VOP states that the radius of the parent vessel dictates the radii of its daughter branches, aimed at maintaining a constant WSS. In line with our findings, Baharoglu et al. previously demonstrated that aneurysmal MCA bifurcations show a significantly lower diameter size ratio (radius ratio) compared to non-aneurysmal bifurcations, which is a violation of the vascular optimality principle^27^.

Another aspect of the optimality principle of work minimization is concerned with the bifurcation angle between parent and daughter vessels^28^. It is suggested that the angles of daughter vessels follow this principle, and that the presence of an aneurysm would be associated with deviations from optimum bifurcation geometry ^5^. We also found larger P1–P1 angles to be associated with BTA presence. These results are consistent with the findings by Lauric et al. that wide bifurcation angles are aneurysmogenic ^29^. In order to control for the possibility that aneurysm formation could affect the surrounding vessel geometry (by increasing the P1–P1 angle), we included a subgroup analysis with small (width ≤ 3 mm) aneurysms only and a sensitivity analysis with the vascular angles excluded, and arrived at similar findings. In a case–control study of 45 patients with BTAs, Tutuncu et al. also found wider basilar bifurcation angles to be significantly associated with the presence of BTAs ^15^. The authors hypothesized that, based on their computational fluid dynamics studies, a wider bifurcation may lead to aneurysm formation by diffusing the flow impingement zone away from the protective medial band region at the bifurcation apex ^15^. Zhang et al. also found 59 BTAs to be significantly associated with wider bifurcation angles, compared to 136 control subjects^14^. However, since their control subjects did not harbor any aneurysms they did not control for possible genetic predisposition and other risk factors.

The main limitations of our study are due to its retrospective design. Aneurysm presence could have affected the morphology of the surrounding vasculature, although we tried to control for this by the subgroup analyses that included only small aneurysms and the sensitivity analyses that excluded angles. Although we included a control group of patients with aneurysms in other locations in order to (partially) control for genetic risk factors, most of these aneurysms are located in the anterior circulation, which may have a different embryological development than posterior circulation aneurysms, possibly with different genetic polymorphisms predisposing to their formation ^30^. All inferences made about the parameters examined can be associated with aneurysm presence only and are not necessarily predictors of formation risk. Measurements were performed manually by a neurosurgeon (JZ) and if needed, verified by a second neurosurgeon (RD). The manual rather than automated analysis may have introduced some variability in the results, but it is a much more applicable technique in the clinical setting. Furthermore, there may be other factors that may be associated with aneurysm formation, such as a history of chronic inflammatory disease and oral infections, that were not taken into account ^31–33^. Finally, the superior cerebellar arteries (SCA) which are proximally located to the basilar tip may contribute to the local hemodynamics and affect the results.

## Conclusions

We examined the contributions of the surrounding vasculature to the formation of BTAs. To minimize confounding by genetic and environmental factors, we included a control group with patients harboring aneurysms elsewhere. We found that P1–P1 angle was significantly associated with BTA presence, whereas diameter size ratio and parent artery diameter were inversely associated with BTA presence. These morphological parameters specific to the basilar apex are practical and straightforward, and support the growing body of evidence that vascular geometry may have a significant effect on aneurysm formation risk beyond other clinical factors. Assessment of these variables when examining 3D-reconstructions of high-risk patients, such as those with a strong family history, could contribute to the risk assessment of these patients.
